# Deformable registration of magnetic resonance images using unsupervised deep learning in neuro-/radiation oncology

**DOI:** 10.1186/s13014-024-02452-3

**Published:** 2024-05-21

**Authors:** Alexander F. I. Osman, Kholoud S. Al-Mugren, Nissren M. Tamam, Bilal Shahine

**Affiliations:** 1 Department of Medical Physics, Al-Neelain University, Khartoum, 11121 Sudan; 2 Department of Physics, College of Science, Princess Nourah bint Abdulrahman University, P.O. Box 84428, Riyadh, 11671 Saudi Arabia; 3 Department of Radiation Oncology, American University of Beirut Medical Center, Beirut, Lebanon

**Keywords:** Deformable image registration, MRI, Convolutional neural networks, Unsupervised learning, Glioma, Neuro-/radiation oncology

## Abstract

**Purpose:**

Accurate deformable registration of magnetic resonance imaging (MRI) scans containing pathologies is challenging due to changes in tissue appearance. In this paper, we developed a novel automated three-dimensional (3D) convolutional U-Net based deformable image registration (ConvUNet-DIR) method using unsupervised learning to establish correspondence between baseline pre-operative and follow-up MRI scans of patients with brain glioma.

**Methods:**

This study involved multi-parametric brain MRI scans (T1, T1-contrast enhanced, T2, FLAIR) acquired at pre-operative and follow-up time for 160 patients diagnosed with glioma, representing the BraTS-Reg 2022 challenge dataset. ConvUNet-DIR, a deep learning-based deformable registration workflow using 3D U-Net style architecture as a core, was developed to establish correspondence between the MRI scans. The workflow consists of three components: (1) the U-Net learns features from pairs of MRI scans and estimates a mapping between them, (2) the grid generator computes the sampling grid based on the derived transformation parameters, and (3) the spatial transformation layer generates a warped image by applying the sampling operation using interpolation. A similarity measure was used as a loss function for the network with a regularization parameter limiting the deformation. The model was trained via unsupervised learning using pairs of MRI scans on a training data set (*n* = 102) and validated on a validation data set (*n* = 26) to assess its generalizability. Its performance was evaluated on a test set (*n* = 32) by computing the Dice score and structural similarity index (SSIM) quantitative metrics. The model’s performance also was compared with the baseline state-of-the-art VoxelMorph (VM1 and VM2) learning-based algorithms.

**Results:**

The ConvUNet-DIR model showed promising competency in performing accurate 3D deformable registration. It achieved a mean Dice score of 0.975 ± 0.003 and SSIM of 0.908 ± 0.011 on the test set (*n* = 32). Experimental results also demonstrated that ConvUNet-DIR outperformed the VoxelMorph algorithms concerning Dice (VM1: 0.969 ± 0.006 and VM2: 0.957 ± 0.008) and SSIM (VM1: 0.893 ± 0.012 and VM2: 0.857 ± 0.017) metrics. The time required to perform a registration for a pair of MRI scans is about 1 s on the CPU.

**Conclusions:**

The developed deep learning-based model can perform an end-to-end deformable registration of a pair of 3D MRI scans for glioma patients without human intervention. The model could provide accurate, efficient, and robust deformable registration without needing pre-alignment and labeling. It outperformed the state-of-the-art VoxelMorph learning-based deformable registration algorithms and other supervised/unsupervised deep learning-based methods reported in the literature.

## Introduction

The current standard conventional deformable registration algorithms and toolboxes, including Syn [1], Elastix [2], advanced normalization tools (ANTS) [3], and demons [4] involve solving a numerical optimization problem independently for each volumetric pair of images by applying geometric methods. This process is usually computationally expensive and time-consuming due to its iterative nature. In addition, these algorithms are non-learning from previous registration and frequently reiterate and optimize. Although the numerical optimization-based image registration methods perform reasonably well, they are restricted by their slow registration speeds.

Deep learning-based techniques have recently received significant attention in medical imaging and cancer treatment e.g. neuro-/radiation oncology [5]. Their capability for the deformable registration task has been increasingly investigated in different medical image modalities [6]. In these methods, in contrast to classical ones, deformable registration is defined as a parametric function. The optimization process is carried out by tuning the learning parameters given a set of fixed and moving images. When the deep learning-based network is trained, it can register a pair of three-dimensional (3D) medical images significantly faster than current standard algorithms [6]. In addition to a significant reduction in the processing time, the recently published deep learning registration methods such as VoxelMorph [7], DLIR [8], and FAIM [9] have demonstrated comparable performance to the standard ones. Based on the way of training the model and the presence/absence of ground-truth data, deep learning-based image registration can be categorized into fully supervised [10], unsupervised [7], and weakly-supervised learning-based methods [11].

Deformable registration of baseline pre-surgical and follow-up volumetric MRI scans is essential to the treatment plan and diagnosis of brain gliomas [12, 13] in neuro-/radiation oncology. Although the decent performance of modern deep learning-based deformable registration algorithms concerning computation time and accuracy, their performance on the brain MR images containing pathologies is far from perfect. This issue remains clinically unresolved. One of the reasons is the high deformation of the brain tissues induced by the resected tumor after the surgery. Some heavy deformations are not restricted to the lesion area and can affect the entire brain. Another reason is that the intensity profiles of the pre-surgical and follow-up scans are inconsistent. A third reason is the lack of correspondence between the pre- and post-operative images [14].

Considering the above points, establishing spatial correspondences between MRI scans acquired at two time-point (e.g., pre- and post-operation) from glioma patients can further help in the understanding of the mechanisms of these tumors. Precisely, for potential tumor recurrence and tumor infiltration, it can further contribute to developing predictive modeling for related pathophysiological processes. It can also aid in understanding the biophysical dynamic and plasticity characteristic of brain tissues besides neurosurgical planning. Few studies have addressed this correspondence utilizing different convolutional neural networks (CNNs) for deformable registration. These methods include proposing a one-stage [15–19], two-stage [20] or three-stage [21] registration pipeline for this purpose. Despite the inspiring performance of these models, obtaining accurate results may remain challenging for deep learning-based deformable registration because of the large deformation of the healthy images. In addition, most of these methods need initial rigid registration performed separately before deformable registration. They involve more than a single stage (two- and three-stage methods) and cannot be fully automated. In this paper, we develop an unsupervised 3D convolutional U-Net-based deformable image registration (ConvUNet-DIR) framework to estimate the correspondences between pre-surgical and follow-up 3D MRI scans for glioma patients. Our method can perform an end-to-end deformable registration (i.e., without human intervention) and does not need supervision (e.g., data such as ground-truth registration fields or anatomical landmarks) during the network training. The network resembles a multi-scale U-Net style architecture [22] to capture the feature maps, and its parameters are updated during the training by minimizing the dissimilarity between the baseline and warped images.

## Materials and methods

**Fig. 1:**
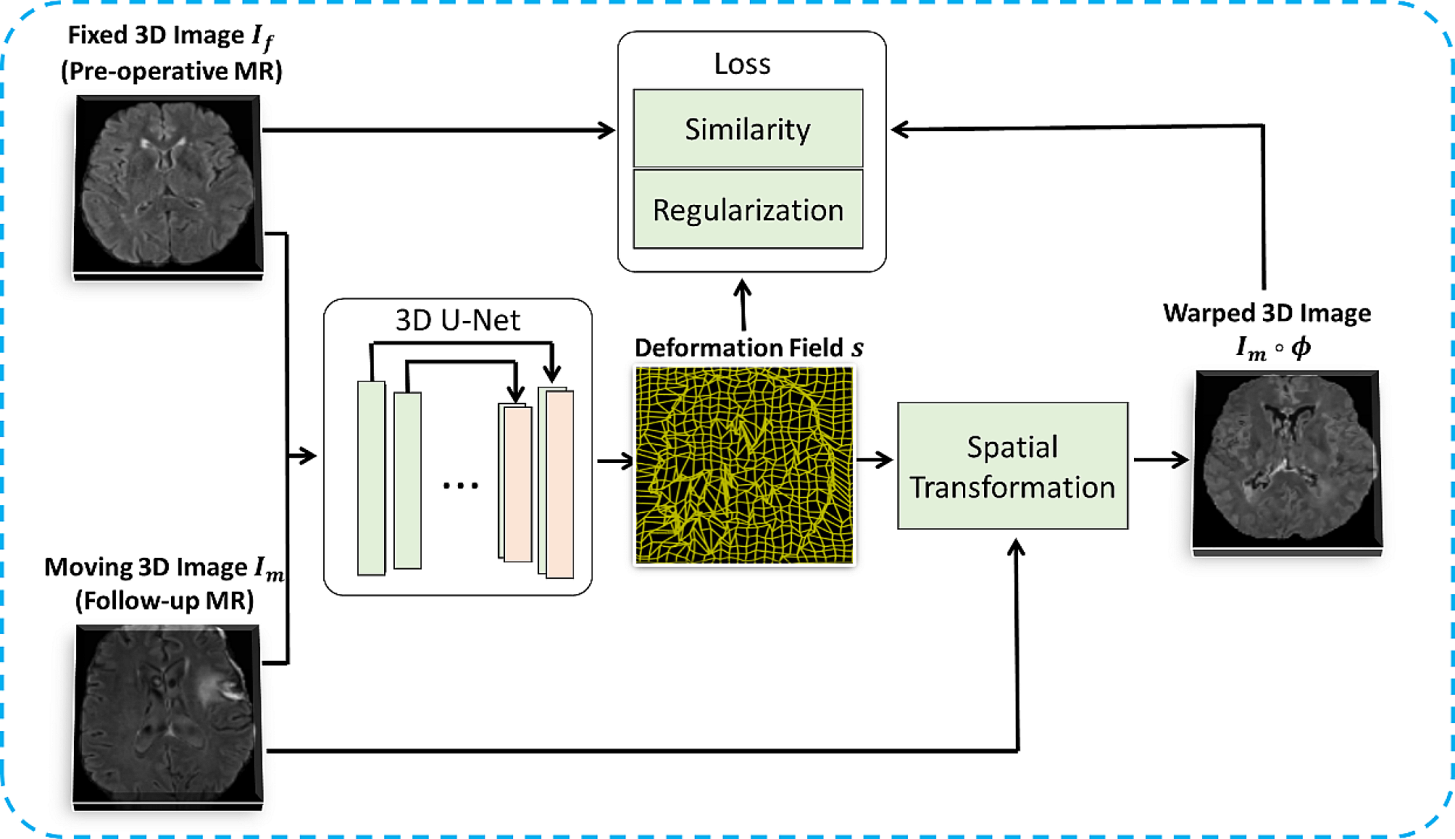
The framework of our unsupervised convolutional U-Net based deformable image registration (ConvUNet-DIR) of pre-operative and follow-up magnetic resonance images of glioma patients

The ConvUNet-DIR framework (Fig. 1) proposed in this study is to estimate the optimal parameterized mapping function $\phi$ between a baseline pre-operative MR image (fixed image, ${I}_{f}$) and the follow-up MR image (moving image, ${I}_{m}$). The $\phi$ is a nonlinear voxel-wise correspondence between ${I}_{f}$ and ${I}_{m}$. The deformed/warped image (${I}_{m}\circ \phi$) from ${I}_{m}$ can be registered to ${I}_{f}$. Here, the global mapping function $\phi \left(x\right) = x + s\left(x\right)$ is formed by an identity spatial transform and deformation field $s$. Once the framework has been trained, the deformation field $s$ can be obtained from a new pair of MRI scans.

### Dataset

A multi-institutional dataset (*n* = 160) of multi-parametric MRI scans, representing the training and validation sets of the Brain Tumor Sequence Registration (BraTS-Reg 2022) challenge, was used in this study [23]. The patients were diagnosed with glioma and clinically scanned with multi-parametric MRI acquisition protocol. The multi-parametric MRI scans of each patient included T1-weighted (T1), contrast-enhanced T1 (T1-ce), T2, and T2 Fluid Attenuated Inversion Recovery (T2-FLAIR or simply FLAIR). The mages were acquired at two time-point: pre-operative (treatment-naïve) and follow-up. The follow-up scans range from 27 days to 37 weeks. The images have been manipulated already. All scans were first transformed into the same coordinate system and then rigidly co-registered to the same anatomical coordinate using the greedy diffeomorphic registration algorithm [24]. Then, the images were skull-striped by extracting the brain tissue and sampled down to 240 × 240 × 155 dimensions with 1 mm^3^ spatial resolution. The brain extraction was performed using the Brain Mask Generator, a deep learning-based algorithm [25]. Precisely, non-cerebral tissues such as the skull, scalp, and dura were removed from all MRI scans.

### Preprocessing

We implemented some preprocessing to the multi-parametric MRI data before being utilized to train the proposed model. *First*, we cropped the multi-parametric MRI data into smaller sizes of 224 × 224 × 155 dimensions by excluding peripheral voxels with no information. *Then*, we resized the data into 128 × 128 × 128 dimensions. *Next*, the image data were normalized using the zero mean and unit variance technique and scaled the data to the [0, 1] range. Accurate registration requires the input MRI scans to be normalized (i.e., voxel intensities range from 0 to 1) to produce consistent results for the images acquired with different scanners/imaging protocols. *Finally*, we randomly split the data into 64% (*n* = 102) as a training set, 16% (*n* = 26) as a validation set, and 20% (*n* = 32) as a test set.

### CNN architecture

**Fig. 2:**
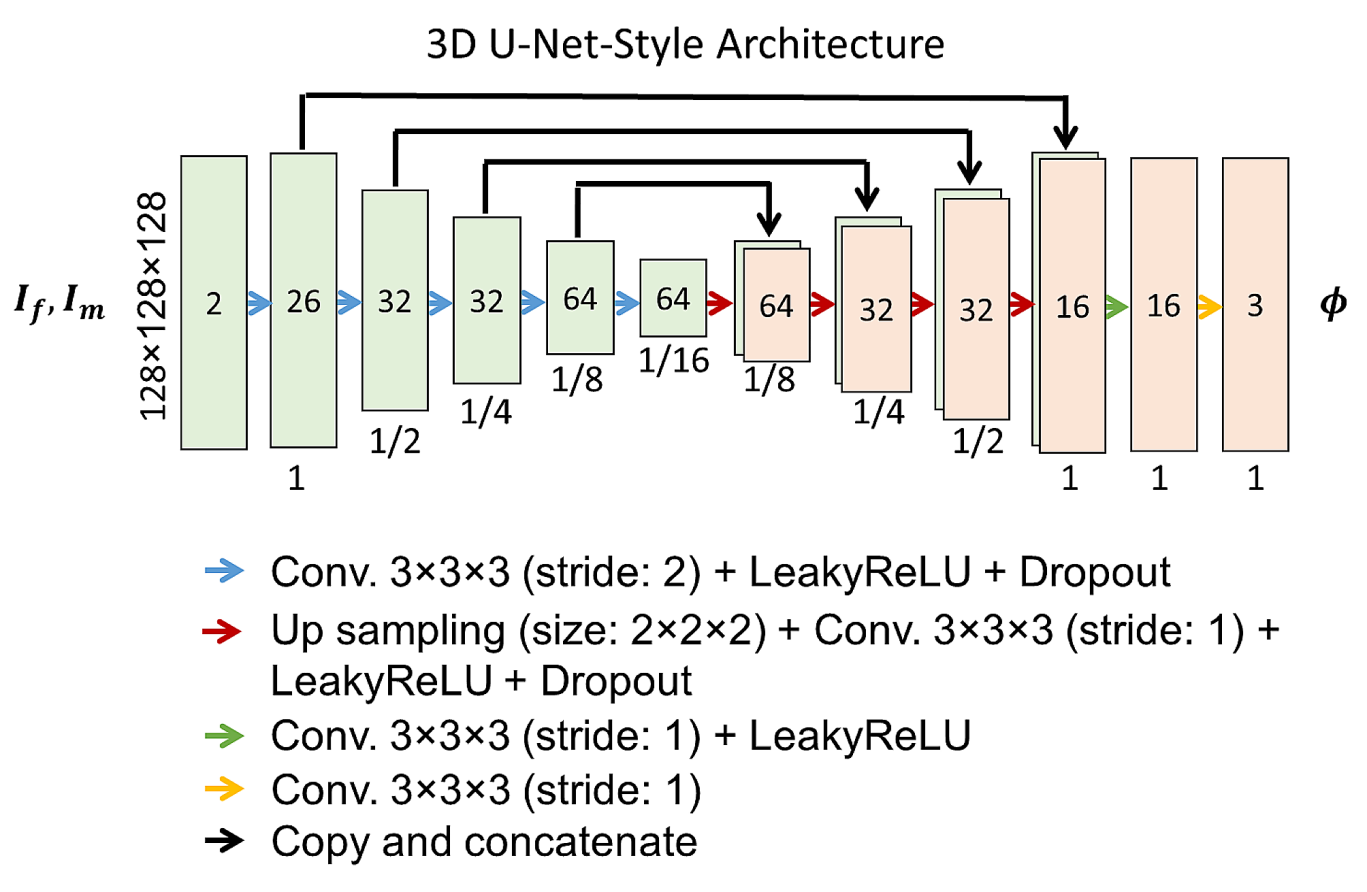
The convolutional U-Net-style architecture. Numbers at the bottom of the blocks represent the spatial resolution ratio of each volume to the input volume. Numbers inside the block indicate the extracted features. Arrows donate different operations

Our proposed ConvUNet-DIR framework uses U-Net [22] as a core (Fig. 2). It takes a two-channel 3D MR image representing the concatenation of ${I}_{f}$ and ${I}_{m}$ as input. The input size of the network is 128 × 128 × 128 × 2. The convolutional network consists of an encoder with four downsampling layers, a bottleneck or bridge, and a decoder network of four upsampling layers. In the encoder, 3D convolutional layers (3 × 3 × 3 kernel size and 2 × 2 × 2 stride) and the LeakyReLU activation are used to extract features and down-sample/reduce the spatial resolution of the volumes by a factor of 1/2 at different spatial resolution levels. The base level of the model or bottleneck is composed of two convolutional layers (3 × 3 × 3 kernel size and 1 × 1 × 1 stride). The decoder network is made up of four upsampling convolutional layers (3 × 3 × 3 kernel size and 1 × 1 × 1 stride) and the LeakyReLU activation function to reconstruct the deformation field. The encoder and decoder networks are concatenated via skip connections to detect and combine information at different spatial levels to produce the deformation field. Three additional convolutional layers (3 × 3 × 3 kernel size and 1 × 1 × 1 stride) with LeakyReLU activation function are added after the decoder network. The final layer is a convolutional layer (3 × 3 × 3 kernel size and 1 × 1 × 1 stride) with a linear activation used for a regression output. The linear activation in this layer enables positive and negative values as the output of the deformation field. The output layer of the network is a deformation field $s$ with a size of 128 × 128 × 128 × 3.

### Spatial transformation layer

The spatial transformation layer computes ${I}_{m}\circ \phi$. The position of individual voxels in ${I}_{m}$ is determined in the space of ${I}_{f}$. In this layer, $\phi$ is used to warp ${I}_{m}$ and then obtain ${I}_{m}\circ \phi$. To ensure that the spatial transformation layer is differentiable, we used linear interpolation to estimate the voxel value of ${I}_{m}$ in the x, y, and z coordinates. As a result, back-propagation of the errors through the network could be implemented during the training.

### Loss function

We implemented an unsupervised loss function ($Loss$) to evaluate the model using only the ${I}_{f}$ and ${I}_{m}\circ \phi$. The loss function consists of a similarity loss (${L}_{sim}$) and a regularization term (${L}_{smooth}$) that penalizes large deformations to produce smooth registration fields. ${L}_{sim}$ penalizes the difference in appearance, whereas ${L}_{smooth}$ penalizes the first derivative of $s$ to produce a fine registration field. The $Loss$ is defined as:

$${Loss\left({I}_{f}, {I}_{m}\circ \phi \right)=L}_{sim}\left({I}_{f}, {I}_{m}\circ \phi \right)+\lambda {\bullet L}_{smooth}({I}_{f}, {I}_{m}\circ \phi )$$

where $\lambda$ is a regularization parameter.

The ${L}_{sim}$ was set to the negative local normalized cross-correlation (NCC) coefficient [26] of ${I}_{f}$ and ${I}_{m}\circ \phi$. This intensity-based similarity measure was found to be optimal for single/mono-modality image registration where the image pair shares similar intensity distribution [2]. The $NCC$ coefficients were calculated over a volume of 9 × 9 × 9. The ${L}_{sim}$ is defined as:

$${L}_{sim}\left({I}_{f}, {I}_{m}\circ \phi \right)=-NCC({I}_{f}, {I}_{m}\circ \phi )$$.

Minimizing the ${L}_{sim}$ without applying constraints can lead to ${I}_{m}\circ \phi$ with unrealistic organ appearances. Obtaining a smooth $s$ requires implementing a diffusion regularizer on the spatial gradients ($\nabla$) of deformation $s$ as follows:

$${L}_{smooth}\left({I}_{f}, {I}_{m}\circ \phi \right)=\sum {\left|\right|\nabla \text{s}({I}_{f}, {I}_{m}\circ \phi )\left|\right|}^{2}$$.

The spatial gradients were approximated using differences between neighboring voxels.

### Training and validation

The ConvUNet-DIR model was trained using an unsupervised manner for deformable registration of a pair of MRI scans (${I}_{f}$, ${I}_{m}$) with volumes of 128 × 128 × 128 on a training data set (*n* = 102). Adam optimizer, implementing the gradient descent approach, was set to optimize the learning model with a 0.0001 learning rate. The regularization parameter, λ, was set to 1. We assessed different settings for λ, including 0 (no regularization), 0.1, 0.2, 0.5, and 1. The λ = 1 found to work best with our task. The model was trained for 150 epochs using a batch size of 1. Our GPU memory does not permit us to use a larger batch size. During the network training, each pair of MRI scans is concatenated into a 2-channel 3D image and fed into the 3D U-Net. The deformation field $s$ was computed through the convolutional layers of U-Net. The spatial transformation layer was used to warp ${I}_{m}$ into ${I}_{m}\circ \phi$ by using linear resampling. The network parameters are regularly tuned during the training by minimizing the dissimilarity between the ${I}_{f}$ and ${I}_{m}\circ \phi$. The $s$ is punished by regularization terms to encourage smoothness (i.e., regularize the predicted deformation). During the training, the model was validated on the validation set (*n* = 26) to assess its generalizability and to update the hyper-parameters.

The training was performed using Keras API (version 2.10) with a Tensorflow (version 2.10) platform as the backend in Python (version 3.10, Python Software Foundation, Wilmington, DE, USA) by using an NVIDIA Quadro M1200 4 GB GPU. The trained model takes less than 2 s to register a pair of MRI scans for a new patient, making its deployment to the clinical practice feasible.

### Evaluation

The model was assessed to evaluate its performance on the registration of pairs of ${I}_{f}$ and ${I}_{m}$ on a test set (*n* = 32). One of the utilized metrics is the Dice similarity coefficient. It is used to estimate the volume overlap of the brain fields which was determined using the generated brain masks. The Dice score of the warped mask (${A}_{m}\circ \phi$) and the fixed mask (${A}_{f}$) of the ${I}_{f}$ is calculated as follows:

$$Dice({A}_{m}\circ \phi ,{A}_{f})=\frac{2|{A}_{m}\circ \phi \cap {A}_{f}|}{\left|{A}_{m}\circ \phi \right|+\left|{A}_{f}\right|}$$

Another metric used to evaluate our proposed method is the structural similarity index (SSIM). This metric simulates the human-perceived quality of images by comparing two images. Mathematically, it is defined as:

$$SSIM(x,y)=\frac{(2{\mu }_{x}{\mu }_{y}+{C}_{1})(2{\sigma }_{xy}+{C}_{2})}{\left({\mu }_{x}^{2}+{\mu }_{y}^{2}+{C}_{1}\right)({\sigma }_{x}^{2}+{\sigma }_{y}^{2}+{C}_{2})},$$

where $\mu$ is the mean image intensity, ${\sigma }^{2}$ is the variance of the image, ${\sigma }_{xy}$ is the covariance of the fixed $\left(x\right)$ and moving $\left(y\right)$ images, and ${C}_{1}$ and ${C}_{2}$ are constants added to stabilize the division with a weak denominator. The performance of the ConvUNet-DIR model was also compared with the open-source VoxelMorph (VM1 and VM2) methods [7], deep learning-based algorithms. We trained the two versions of the VoxelMorph algorithms from scratch on the BraTS-Reg 2021 dataset for a fair comparison.

## Results

**Fig. 3:**
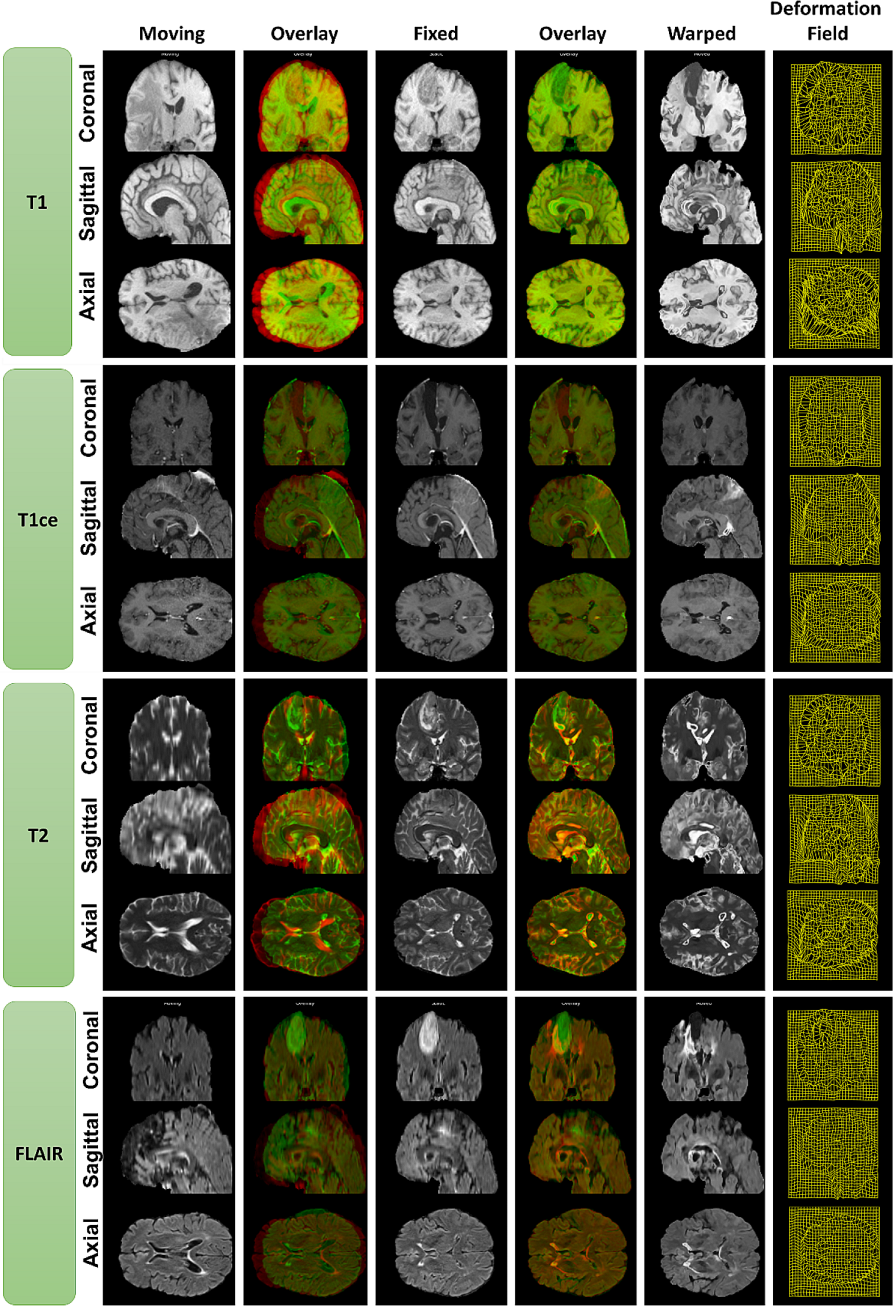
Example registration results of pairs of T1, T1-ce, T2, and FLAIR 3D MRI scans on three planes (axial, coronal, and sagittal) by our proposed ConvUNet-DIR model for one patient in the test set. Overlays of the fixed image (green color) and moving/warped image (red color) on top of each other and the deformation field are also displayed

**Fig. 4:**
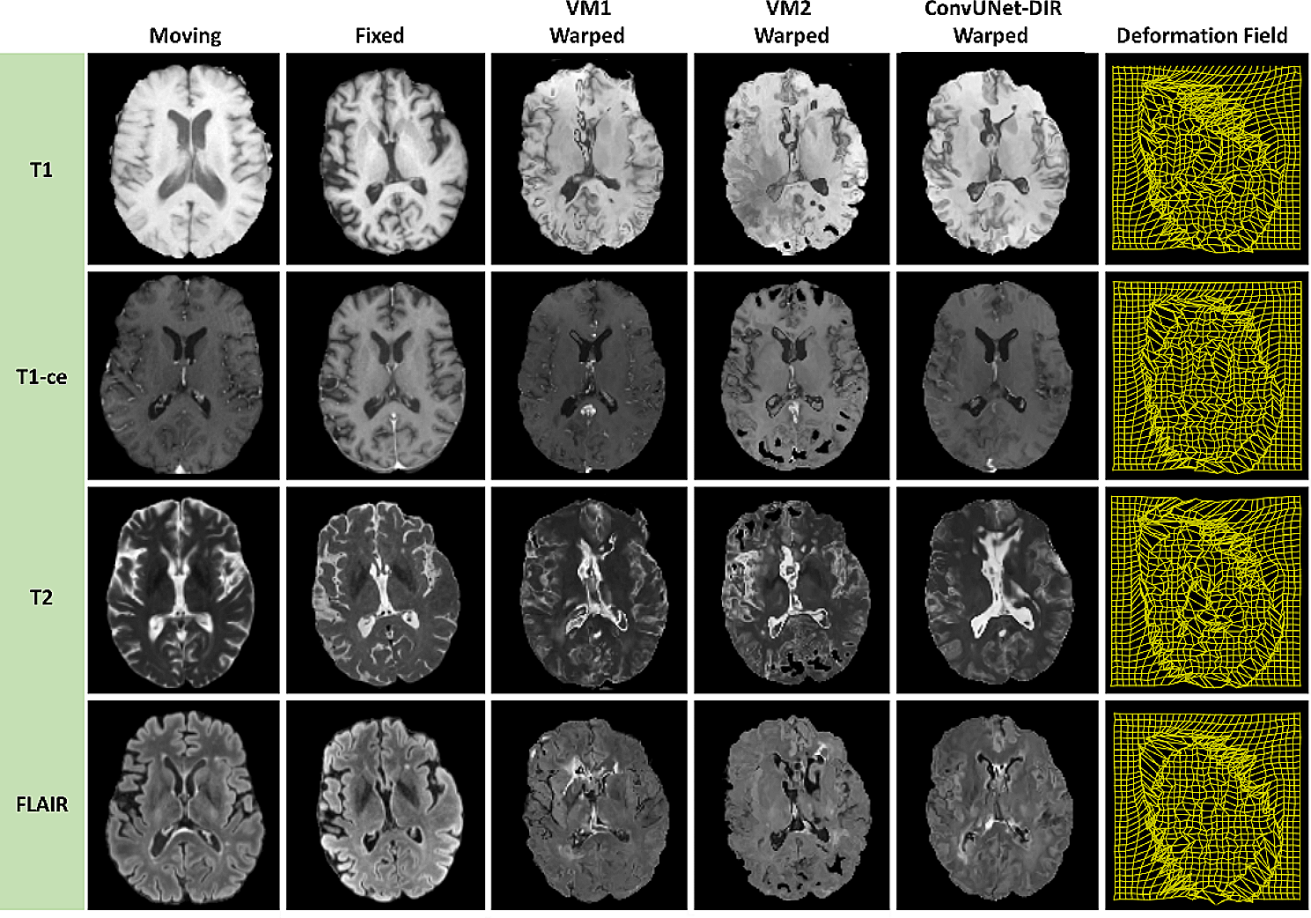
Example registration results of pairs of T1, T1-ce, T2, and FLAIR 3D MRI scans with the ConvUNet-DIR and VoxelMorph (VM1 and VM2) models

The results of the ConvUNet-DIR model are reported for pairs of T1, T1-ce, T2, and FLAIR MRI scans on the test set (*n* = 32). Figure 3 shows representative registration results of aligning two images for one patient on axial, coronal, and sagittal views. The figure also displays the overlay of the fixed (pre-operative) and moving (follow-up) images, the overlay of the fixed and warped (deformed) images, and the deformation field. The overlay of the fixed and moving images shows variable degrees of deformation, whereas the warped image is nearly overlapping on the fixed image. The figure shows the fixed image (in green color) on top of the moving image (in red color) and the warped image (in green color) on top of the fixed image (in red color). It looks like the deformed image has a slightly lower spatial resolution. The results of the deformable registration of the four MRI scans with ConvUNet-DIR and VoxelMorph (VM1 and VM2) models are shown in Fig. 4. It is clear that the ConvUNet-DIR model provides better registration results compared to both VM1 and VM2 algorithms.

**Fig. 5:**
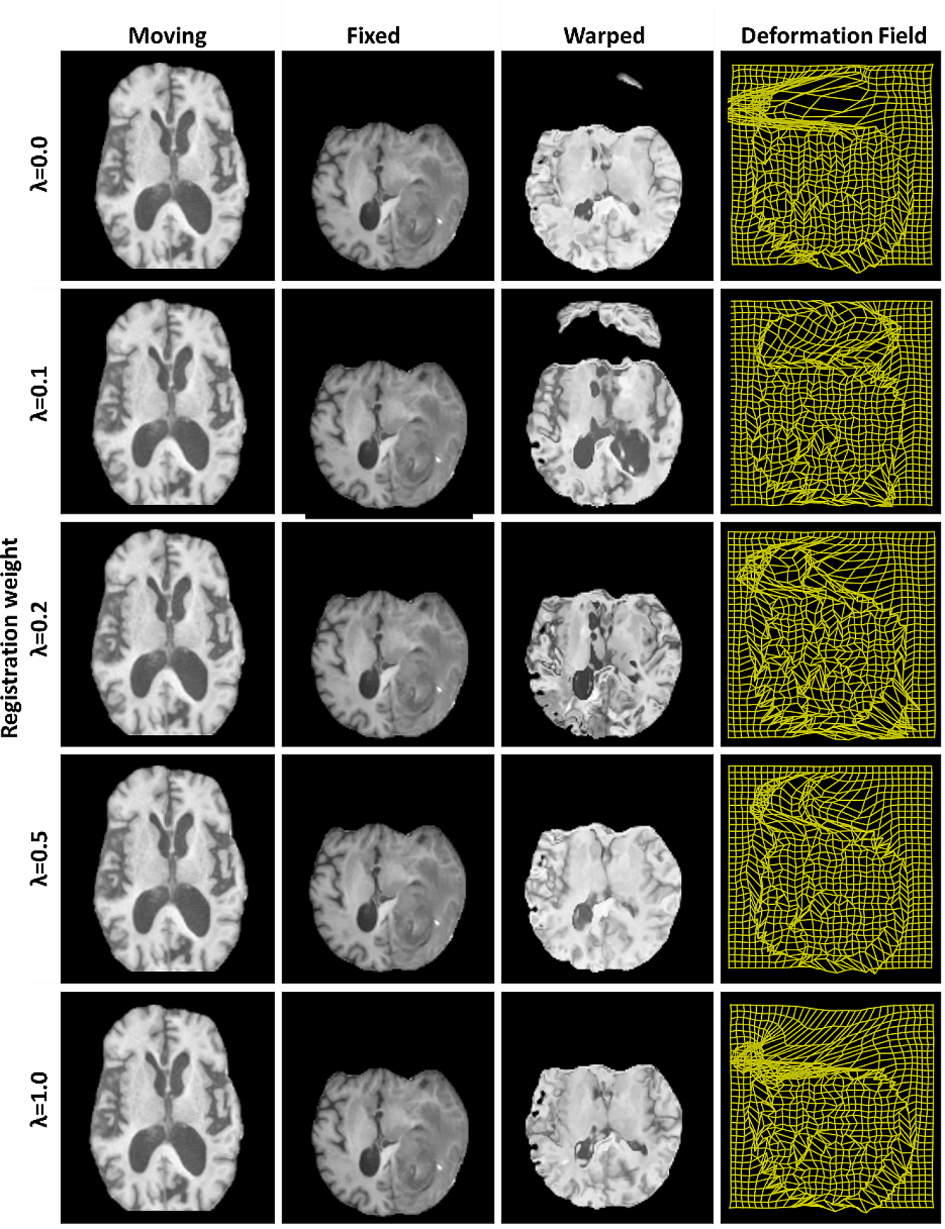
Results of effects of the regularization parameter, λ, on the warped image (T1 as an example) produced by the ConvUNet-DIR model. Rows show different regularization weights. Columns show the moving image, fixed image, warped image, and the deformation field, respectively

Figure 5 shows the effects of the regularization parameter, λ, on the warped image (T1 as an example) generated by the ConvUNet-DIR model. The results demonstrate that the best registration is obtained with the regularization weight set to 1, which agrees with that reported by Chen et al. [27].

Table 1 presents a quantitative summary of all registration results with the ConvUNet-DIR, VM1, and VM2 models on the test set (*n* = 32). The mean SSIM and Dice scores were reported for all correspondences. The SSIM and the Dice score were calculated for the brain fields of a 3D MR image. From the results, we can observe that the performance of the ConvUNet-DIR method is consistently better compared to VM1 and VM2. The table also reports the CPU computation time required by each model to perform deformable registration on a pair of volumetric MRI scans for a new patient. The computation time of all models was about 1 s, which is significantly shorter than the traditional methods. The learning parameters of all models also were reported in the table.

**Table 1:** Comparison results of the proposed model (ConvUNet-DIR), VM1, and VM2 models for deformable registration of pairs of 3D MRI scans evaluated on a testing data set (*n* = 32). Results were reported in the form of mean ± 1 standard deviation. Values in bold indicate the best results

Modality	Model	SSIM	Dice Score	CPU (s)	Parameters
T1	VM1	0.876 ± 0.052	0.965 ± 0.007	0.83 ± 0.17	259,675
	VM2	0.818 ± 0.120	0.943 ± 0.015	1.34 ± 0.22	300,547
	ConvUNet-DIR	**0.898 ± 0.041**	**0.976 ± 0.005**	1.32 ± 0.14	328,227
T1-ce	VM1	0.906 ± 0.039	0.974 ± 0.008	0.83 ± 0.17	259,675
	VM2	0.848 ± 0.104	0.959 ± 0.012	1.34 ± 0.22	300,547
	ConvUNet-DIR	**0.919 ± 0.035**	**0.980 ± 0.008**	1.32 ± 0.14	328,227
T2	VM1	0.875 ± 0.053	0.962 ± 0.010	0.83 ± 0.17	259,675
	VM2	0.816 ± 0.118	0.937 ± 0.018	1.34 ± 0.22	300,547
	ConvUNet-DIR	**0.894 ± 0.043**	**0.971 ± 0.008**	1.32 ± 0.14	328,227
FLAIR	VM1	0.878 ± 0.046	0.964 ± 0.010	0.83 ± 0.17	259,675
	VM2	0.821 ± 0.113	0.945 ± 0.022	1.34 ± 0.22	300,547
	Ours	**0.896 ± 0.041**	**0.973 ± 0.008**	1.32 ± 0.14	328,227
Overall (T1, T1ce, T2, & FLAIR)	VM1	0.884 ± 0.011	0.966 ± 0.004	0.83 ± 0.17	259,675
	VM2	0.826 ± 0.011	0.946 ± 0.006	1.34 ± 0.22	300,547
	ConvUNet-DIR	**0.902 ± 0.009**	**0.975 ± 0.004**	1.32 ± 0.14	328,227

SSIM: structural similarity index; VM1: voxelmorph1; VM2: voxelmorph2

## Discussion

Deformable registration of MRI scans of patients with pathologies is challenging. In this study, we developed an unsupervised deep learning-based deformable registration algorithm to establish complex correspondences between the pre- and post-operative 3D MRI scans of patients with glioma. Given a pair of MRI scans at two time-point as input, the ConvUNet-DIR model computes the voxel-wise deformation between the two images. In addition to its 3D nature that accounts for adjacent slices in the volumetric image data, ConvUNet-DIR does not need supervision during its training.

The qualitative results are illustrated in Figs. 3 and 4 for an example patient in the test set. The ConvUNet-DIR method demonstrated registration with high accuracy while preserving deformation smoothness. It also seemed to preserve the original intensity distribution better, while VM1 and VM2 appeared to have an impact on the intensity values. The quantitative registration results are presented in Table 1. The table shows impressive results achieved by our proposed model with a mean SSIM of 0.908 and a mean Dice score of 0.975. The execution time of the ConvUNet-DIR model was about 1 s, which is significantly shorter than the conventional methods. This advantage signifies its clinical deployment for critical time applications in neuro-oncology and radiation oncology.

This study also compared the performance of the ConvUNet-DIR with the VoxelMorph models [7], as shown in Table 1. Both methods are deep learning-based algorithms that allow unsupervised 3D deformable registration. The results achieved by ConvUNet-DIR (SSIM = 0.908, Dice = 0.975) were superior to VM1 (SSIM = 0.893, Dice = 0.969) and VM2 (SSIM = 0.857, Dice = 0.957). Our method produces a more competitive deformation field than VoxelMorph. We also compared the performance of the ConvUNet-DIR with the current state-of-the-art supervised and unsupervised deep learning-based approaches reported in the literature utilized public brain MRI datasets. Our method (Dice = 0.980) outperformed Han et al. [19] (Dice = 0.839), Martin et al. [28] (Dice = 0.756), Wu et al. [29] (Dice = 0.873), Meng et al. [30] (Dice = 0.654), Kuang and Schmah [9] (Dice = 0.533), Mok and Chung [31] (Dice = 0.770), Huang et al. [32] (Dice = 0.707), Xu et al. [33] (Dice = 0.830), Dey et al. [34] (Dice = 0.781), Fan et al. [35] (Dice = 0.788), Liu et al. [36] (Dice = 0.909), Chen et al. [37] (Dice = 0.873), Wang et al. [38] (Dice = 0.731) methods using unsupervised approach; and Zhu et al. [39] (Dice = 0.637) method using supervised approach.

This study has demonstrated that the proposed method can perform an end-to-end deformable registration of a pair of volumetric brain MR images without human intervention. It does not require pre-alignment compared to similar algorithms that require at least two steps to perform the registration [20, 21]. Compared to supervised registration networks, our method does not require additional segmentation masks or anatomical marks for network training. ConvUNet-DIR could also support multimodality image registration by substituting NCC with mutual information. Combining T1 pre- and post-contrast, T2, and FLAIR might achieve even better registration. The reason is that different scans are implicitly co-registered within the pre- or post-operative data (just some rigid registration), barring image distortion from the acquisition). It is worth noting that ConvUNet-DIR works for a broad window of time (27 days to 37 months follow-up).

We can briefly discuss the limitations of this study in three points. First, our model requires resizing all images, which may cause information loss. Employing a tri-linear interpolation could minimize this issue. Second, due to the restriction of the GPU memory, we set the batch to only 1 sample. Using a small batch size may cause registration errors. Third, this work is a multi-institution study with a relatively small dataset. To assess the performance of this model in a more general way in clinical practice, we recommend training the model using more data samples from several institutions for better generalizability.

## Conclusions

We developed a ConvUNet-DIR framework based on unsupervised learning to establish correspondence between a pair of 3D MRI scans acquired at two time-point from patients with glioma. The proposed method demonstrated registration accuracy superior to the state-of-the-art VoxelMorph (VM1 and VM2) registration tools (open-sourced learning-based registration algorithms) and other supervised/unsupervised deep learning-based algorithms reported in the literature. It can perform an automated deformable registration of a pair of 3D MRI scans for glioma patients. The model could provide accurate, efficient, and robust deformable registration without needing pre-alignment and labeling, resulting in a significantly shorter registration time. This method has the potential for application in clinical practice in neuro-/radiation oncology.

## Data Availability

The datasets generated during and/or analyzed during the current study are available at the Brain Tumor Sequence Registration (BraTS-Reg) Challenge repository (https://www.med.upenn.edu/cbica/brats-reg-challenge/).

## Abbreviations

3D Three: dimensional
ANTS: Advanced normalization tools
BraTS-Reg 2022: Brain Tumor Sequence Registration 2022 challenge
CNNs: Convolutional neural networks
ConvUNet-DIR: Convolutional U-Net-based deformable image registration
MRI: Magnetic resonance imaging
NCC: Normalized cross-correlation
SSIM: Structural similarity index
T1: T1-weighted
T1-ce: T1-weighted with contrast enhancement
T2: T2-weighted
T2-FLAIR: T2-weighted Fluid Attenuated Inversion Recovery
VM1: VoxelMorph1
VM2: VoxelMorph2
